# The Selection of Consumptive Cases for Sanatorium Treatment

**Published:** 1902-10-04

**Authors:** T. N. Kelynack

**Affiliations:** Assistant Physician, Mount Vernon Hospital for Consumption and Diseases of the Chest, Hampstead and Northwood, London


### The Selection of Consumptive Cases for Sanatorium Treatment.

By T. N. KELYNACK, M.D., M.R.C.P., Assistant Physician, Mount Vernon Hospital for Consumption and Diseases of the Chest, Hampstead and Northwood, London.

Consumption, at least in the mind of the public, has for long been synonymous with a hopeless and helpless despair. The diagnosis of consumption once definitely pronounced, from the standpoint of the sufferer's friends amounted to a virtual signing of the death certificate; and such a view was oftentimes endorsed by the physican. But the old pessimism is passing. The pathological evidence of the curability of phthisis is abundant and the clinical witnesses are no less convincing. The *Spes phthisica* is no mere will-o'-the-wisp; it rests on a sound psycho-physiological basis.

Consumption has proved itself a disease of every age, every race, and every land. It is co-extensive with the habitable regions of the earth. With uncertainty as to its causation the nations may well have faced fate with folded arms.

But the epoch-making discovery of Koch, in 1882, has changed all that. The recognition of the excitant has aroused hope and quickened energy. The unmasking of the seed has led to earnest and scientific investigation of the soil. The scientific basis of the work of such pioneers as Bodington, MacCormack, and Bennet in this country, and Brehmer, Walther, and others in Germany, has now been recognised and abundantly acknowledged. The gospel of hygiene has revealed not only means for the maintenance of health, but rational measures to secure the salvation of the sick. We now know that such hygienic requirements as are necessary for the healthy are even more essential for the diseased.

Although we have been long groping in the dark, fettered by ignorance, apathy, and superstition, sure progress has been made, and that along a right road. Dr. Ransome has shown that consumption in this country is now much less prevalent than formerly. In 1838 the phthisis rate of mortality per 10,000 was over 38; in 1895 it was nearly 14, a diminution by almost two-thirds. Dr. Ransome, writing in 1898, sought to show that if consumption continued to diminish at the same rate during another 30 years, it would have entirely disappeared by the end of that period. Unfortunately the extermination of this disease is not to be gauged by mathematics or foretold by prophecy. It is probably destined long to linger as an evidence of human frailty and national perversity. But it is important to note that the lessening in the prevalence of phthisis has coincided with public improvement in sanitary affairs. Further progress is now being achieved by personal reform and a greater individual attention to hygienic considerations. There is also reason to believe that slowly and silently important changes are occurring both in the nature of the tubercular seed and the character of the tubercular soil. The degree of virulence of the tubercle bacillus undoubtedly varies greatly with different cultures. And certainly the degree of predisposition to infection varies immensely. Many persons constantly exposed to the risk of infection pass unscathed. Some individuals, like certain animals, seem practically immune. The improved conditions of human life, and the more perfect physical development of a large proportion of the younger members of the community, undoubtedly make for a more strenuous and successful resistance. The average duration of life in phthisis is without doubt longer than was formerly supposed. Even in the absence of all treatment the process may be arrested, as has been conclusively proved by the evidence of the post-mortem room. And with a return to rational methods of treatment life is being lengthened for even advanced cases, and the disease arrested in a large proportion of instances; and many are so "cured" as to be restored to a condition compatible with fairly long life.

The pessimism of the past led to supineness, apathy, and despair. The optimism of the present stimulates to early, vigorous, and efficient action. It is now necessary not only to recognise the earliest indications of disease, but to warn those who, from inherited or acquired conditions, are predisposed to infection. The fetters of old ideas lie broken. The bonds of a mistaken pathology have been loosed. Quietly and almost unnoticed rapid advance has been made, until even the mind and heart of the sleepy, easy-going, fickle public have been conquered, and all stand eager to lend a hand in the extermination of "the great white plague."

### A Comprehensive Campaign.

The *hygienic treatment of consumption* has now been clearly established as the only rational way to cope with this scourge. This can best be carried out in suitably situated and properly conducted establishments under strict medical control. In all civilised countries the sanatoria movement is now acknowledged as affording the most scientific and effective means of combating consumption. But discouragement and bitter disappointment must result unless it be realised that the establishment of sanatoria is not the beginning and the end of the matter. It forms only a link, although a very important and necessary link, in the strong chain which must be forged if consumption is to be effectually fettered. The profession and public must realise that we have entered on *a comprehensive campaign.*

We are but at the beginning of the war. The weapons are new, or perhaps it might be better said those who wield them are inexperienced. We are still to a great extent in the stage of experiment. Hitherto the warfare has been too desultory. The need for federation, co-operation, and combination is pressing. A new principle has been grasped, but much work has to be accomplished before satisfactory details are arrived at.

In the conduct of a comprehensive campaign against consumption the following are requisite:—

1. The recognition and protection of all persons predisposed to tubercular infection.
2. The detection of the earliest manifestations of consumption.
3. Notification of all consumptives.
4. Private sanatoria or effective home treatment for the affluent.
5. Public dispensaries or out-patients' departments.

6. Public hospitals, situated on the borders of towns and cities, for observation of patients and treatment of acute cases.

7. Public sanatoria, situated in the country, but if possible readily accessible.

8. Seaside sanatoria for children.

9. Convalescent homes for consumptives.

10. Homes for advanced cases.

11. Country colonies for "arrested" cases.

12. State hospitals, sanatoria, and homes for the dying for all destitute cases.

It is impossible within the confines of this short paper to enlarge on the various points which need consideration in the presentation of such a programme.

But stated briefly provision has to be found for *three classes of patients*:—

1. The *affluent* or well-to-do may secure not only efficient hygienic treatment, but even non-essential luxuries in the many *private sanatoria* which have sprung up in this country. They may journey to various foreign climatic stations; or effectual treatment may be provided in their own country houses, although in such cases the discipline and medical supervision which constitutes such an important element in sanatorium treatment is in danger of being lost. They will in all cases of course be directed by their medical advisers.

And here in passing the hope may be expressed that medical men will not allow themselves to be in any way connected with institutions which merely seek financial advantage by the exploitation of the so-called "open-air" treatment. Such can only bring disappointment and much discredit to sanatorium treatment.

2. But a large number of consumptives belong to the *competent* class, that is to say those who while paying their way and living in relative comfort cannot afford to meet any prolonged financial strain on their resources. In this class we find young professional men, civil servants, small tradesmen, curates, clerks, farmers, nurses, many shop girls, governesses, and the like.

For these Public Sanatoria are essential. Their maintenance must at least in great part depend upon the support of the benevolent. In some instances part payment can be afforded. Possibly a method of deferred payment in case of cure would meet the wishes of some.

Public charity, unless better organised or more abundant, must fail to make adequate provision for this very numerous class. The available establishments are all too few. The number of applicants waiting admission are all too many: they are to be counted by the hundreds. The period of residence is miserably meagre. In the selection of cases most suitable for admission many difficulties, perplexities, and discouragements arise. The selection at the present time has to be made in great measure according to a method based on expediency.

3. For the great bulk of the *indigent*, the poor drawn from the artisan and labouring class and including workmen, domestic servants, seamstresses, and many girls employed in shops and factories, State Sanatoria should be provided out of the public funds.

For a large proportion of the cases now flooding the out-patient departments of our general and special hospitals, the only and yet much dreaded refuge is the Poor Law Infirmary or Workhouse Hospital. Much in connection with our present poor law administration of relief to the sick is repugnant and particularly repellant to those afflicted with such a disease as consumption. In most cases facilities for the suitable care of such cases are entirely deficient or quite inadequate. The public intelligence and conscience need to be aroused in this matter. As Sir James Crichton-Browne has well contended, State sanatoria for the poor consumptive must be kept "free from any taint of pauperism, and have no point of contact with the Poor Law." It would be well if the establishment and management of such sanatoria should devolve on the County Councils, acting singly or in combination. And it should be clearly understood that the extermination of consumption is impossible as long as the poor and therefore necessarily more or less neglected and advanced consumptive, is allowed to linger and die in his over crowded insanitary odging.

### Principles of Sanatorium Treatment.

In selecting a suitable case for sanatorium treatment it is well to remember what hygienic management seeks to secure:

I. The removal of the patient from fresh invasion by the tubercle bacillus, and separation, as far as possible, from all influences aiding its introduction.

II. The establishment in the patient of processes of repair and the development of the highest powers of resistance.

To this end the instruments employed are:

1. Continuous exposure to fresh air.
2. Free access to sunlight.
3. Regulated rest.
4. Controlled exercise.
5. Abundant feeding.
6. Obedience to hygienic requirements.
7. Strict medical supervision.

It must be clearly understood that sanatorium treatment offers no specific for the cure of consumption. It seeks to encourage and ensure the exercise of those natural powers which, fortunately, in a large proportion of cases, strongly make for restoration. And these benefits depend on carefully regulated regime and selected environment. But amidst humanitarian, professional, and financial considerations it is not always easy to steer a clear course.

It must be admitted at once that no absolute rules can be laid down for the selection of cases. He who seeks to be guided by a code will quickly find himself blundering. Every case must be the subject of serious study. In forming an opinion cumulative evidence is necessary. The too cautious and the too reckless will soon find themselves companions in the same ditch. Disappointment must sometimes be the portion even of the most careful and unexpected fortune will occasionally wait on the least hopeful. It is much to be deplored that through the exigencies of the present situation many a doubtful case must forego "the only chance."

In many cases "risks" must be accepted. He who risks nothing gains nothing. Even the most favourable case may suddenly pass over into the category of unpromising or even seemingly hopeless ones by the occurrence of such complications as hæmoptysis, pneumothorax, acute pleurisy, septic infection, recrudescence or extension of the tuberculous process. It is therefore only possible to indicate in bare outline, and that most tentatively, what may be considered the chief evidences on which our verdict must be based.

### The Selection of Cases for Sanatorium Treatment.

Although much has been written concerning the requirements of sanatorium regime and endless discussion has ensued as to the relative value of the various factors concerned in the open-air treatment, comparatively little has been written regarding the selection of cases. The subject is certainly peculiarly difficult. Adequate material for the formation of an absolutely reliable opinion is not forthcoming. Our rules for guidance are therefore merely provisional.

No doubt nearly all cases of consumption would receive benefit by judiciously applied sanatorium treatment. Undoubtedly many advanced cases are much alleviated by the adoption of "open air" methods. And even for apparently hopeless cases hygienic measures often bring a certain measure of relief.

But as I have already shown, the demand for sanatorium treatment far outruns the supply. Indeed, efforts to meet the incessant and piteous appeals for assistance have necessitated the adoption of measures which secure little more than the minimum of benefit. At present cases have often to be kept waiting in a most unsuitable and highly prejudicial environment for many weeks, and not infrequently for several months, before entrance to a sanatorium can be arranged. The six weeks to three months allowed for residence in such sanatoria are all too short to accomplish more than the beginnings of benefit. And yet, in spite of all, incalculable good is being accomplished. In many cases partial or complete restoration to health is attained. And here I would urge that it would be well if all medical officers of sanatoria would agree not to register any case as "cured" unless two years after leaving the sanatorium there is no evidence of activity. In many instances also patients greatly profit by the hygienic instruction received and continue methods making for repair after returning to their own homes. And on the patient's friends and the public generally a beneficent educational influence has been brought to bear. But if the system is to be kept from falling into disrepute, and the greatest good extended to the greatest number, more care must be taken in the choice of cases.

At the present time the selection for sanatorium treatment must evidently be largely influenced by the position and resources of the patient. Before recommending institutional treatment for a sufferer whose means are limited the physician has to bear in mind many considerations; whereas, for the patient who is well off the decision is determined mainly, if not entirely, by the medical aspects of the case.

Although in this paper I am dealing chiefly with the question of the hygienic treatment of consumption as applied in special sanatoria, it is most necessary to insist that there is nothing specific about such treatment. Sanatoria may be good, bad or indifferent according to the manner in which hygienic measures are rationally applied. For many patients in all ranks of society a sanatorium offers not only the most advantageous conditions for securing the establishment of processes making for "natural cure" but also provides a very practical demonstration of the benefits of a hygienic life.

But it is well for the practitioner to realise that even when financial considerations do not bar the way to the selection of the best sanatorium wise discrimination is needed in the choice both of a case and an institution. Prevailing fashions must not be blindly followed. It must be remembered that for the well-to-do at least residence in a sanatorium is only one of several courses which may be adopted. But for all consumptives hygienic treatment is essential. Its precise method of application may be to some extent governed by circumstances. It is sometimes urged that cure can be best attained and should be undertaken under those climatic conditions which the patient must meet when recovery has been secured and the customary avocation is again taken up.

But while there is apparently no place on earth where consumption may not occur and likewise probably no place where consumption may not be "cured," the advantages of a judiciously-selected climate in the management of consumptives must not be minimised. A good climate secures those conditions which are essential for the full maintenance of a hygienic life. No climate is perfect, but residence in certain districts presents special difficulties in obtaining any adequate approach to ideal conditions. It is sometimes possible only by change of residence to select such as will permit of a patient following with comfort the hygienic life essential for restoration and the prevention of relapse. Before recommending residence abroad, the practitioner should always make himself fully acquainted not only with the medical aspects of the case but with the general circumstances of the patient.

Much discrimination is necessary in making the the best arrangements for the well-to-do consumptive. Undoubtedly residence in Alpine sanatoria is peculiarly advantageous for many. For others a warm and dry district at a low level is best. The selection must be made in accordance with the results of experience and not merely based on theoretical considerations.

A sea voyage was in bygone days frequently recommended as a means of coping with early phthisis. In not a few instances good results were secured. But the risks were great, the discomforts might be many, and the advantages were often few. It is now generally recognised that for the consumptive the drawbacks inseparable from ocean travel far outnumber any possible benefits. Yet it must be admitted that under certain circumstances and for particular cases a sea voyage may offer excellent hygienic conditions for pulmonary repair. But as a rule a ship constructed and conducted for the travel of the healthy cannot be expected to serve as a perfect sanatorium for the sick.

In arranging for the so-called hygienic treatment of consumption the practitioner must take a wide outlook. For some patients a fully organised sanatorium treatment is particularly desirable. For others, and especially where the disease has arrived at a stage of quiescence, a comparatively short residence in a sanatorium will secure such training in hygienic procedures as will allow of the following of some form of work in the open air, such as farming, estate managing, rent collecting, or even car or cab driving, and the like.

For some patients unfortunately one can only aim at providing comfort and not cure. For this class much relief will often come from a carefully selected climatic station, such as may be found along our south coast or on the Riviera.

If progress in our methods of treating consumption is to be maintained it will be very necessary to avoid the restricting and stultifying influence of any mere system and adopt only such rational procedures as are based on a recognition and application of natural laws.

### The Public Sanatorium.

It is particularly difficult to formulate conditions for admission to a public sanatorium. Some have insisted that only those patients should be passed who are presumably curable. In all instances a careful report should be supplied if possible by the practitioner who has had opportunities of watching the case. In every instance a period of probation should be allowed during which the patient is under strict observation. Time and tact are needed in many cases to elicit data which may form the basis for a reliable opinion. For certain public sanatoria it has been proposed to submit all reports concerning applicants for treatment to a medical committee. But such a course presents objections and many difficulties.

In accepting the part of medical adviser to a sanatorium the practitioner undertakes a heavy responsibility. His opinion should be based on sound pathological knowledge and wide clinical experience. It is an unkindness to a patient, and an injustice to an institution, and a wrong to scientific medicine, to recommend any case which cannot be permanently benefited.

With ordinary care and thoroughness in examination the diagnosis of phthisis is comparatively easy, but the formation of a satisfactory prognosis is often fraught with difficulties and encircled by pitfalls. It is well to remember that the evidences of constitutional disturbance often precede the development of local manifestations. The practitioner must be alert to recognise the very first indications of mischief. When well-marked physical signs are apparent the "incipient" stage must be considered as passed. The old pessimistic views of the inveteracy and incurability of consumption still linger. Patients still delay in seeking the confirmation of their worst fears. And sometimes, even yet, it is to be feared practitioners fail to take those steps which would secure the prompt adoption of hygienic measures adequate to arrest the morbid process in the earliest stage. Progressive loss of weight, anæmia, lack of vigour and listlessness, vague cough, doubtful blood spitting, indefinite "stitch in the side" and the like are indications of a deviation from health which should always demand thorough medical investigation.

It is most desirable that when a medical man undertakes the responsibility of advising a patient to seek admission to a sanatorium, and certifies that such a case is suitable, that opinion should be the outcome of a serious study of the case. Particulars concerning the family vitality and predisposition to disease, the social conditions of the sufferer, previous illnesses and the development of the present state should all be carefully ascertained. Systematic and continuous observations for at least a week should be made as to temperature, pulse, and respiration. Much attention should be given to the general constitutional state, and a full and definite statement as to the local pulmonary conditions. In fact, a complete report should be available for the guidance of the medical staff of every sanatorium. Surely it is reasonable to expect that at least as thorough an investigation should be made as is demanded by a high-class insurance office.

Every physician, having experience of an out-patient department, well knows the immense amount of extra work often devolving upon him in filling up certificates for convalescent homes. According to our present happy-go-lucky English methods, these forms are usually filled up in a more or less formal manner; and it is to be feared in a like spirit many certificates for entrance into a sanatorium are prepared.

In the selection of cases the necessities of the situation require that economic as well as clinical considerations should have their part. Patients able to bear the full expense of private sanatorium treatment, of course, have no right to seek entrance to an institution supported by public charity.

Poor Law cases should also be excluded. Indeed, under present circumstances, I fear it is no kindness to admit a case into a public sanatorium when we know that in three months, or before, the patient must of necessity pass to the workhouse hospital.

The exigencies of the present situation make it almost necessary to exclude all poor aliens. Provision for these cases, as already indicated, should be met at once by the establishment of suitable State sanatoria.

Acute and doubtful cases should be admitted to a public hospital for consumption, conducted in accordance with strict hygienic methods, and situated on the borders of the town or city.

A system which would allow of a patient being admitted "on probation" would serve to meet the needs of many a doubtful case.

Many general hospitals are now closing their doors on these sad sufferers. It would be well if, for the present, and until the establishment of a larger number of suitable sanatoria, these institutions could set aside one or more wards solely for consumptive patients.

### Cases Suitable for Treatment in Public Sanatoria.

In selecting suitable cases for treatment a definite aim must be kept in mind. It is that under the given circumstances not merely arrest is to be procured but the patient must be firmly placed on the high road to definite cure. A disappearance of the constitutional symptoms must be obtained and a return of normal energy secured. In short, the patient should be fitted to successfully take his place again as a bread-winner.

At present it is absolutely necessary to limit admission to such cases as may be expected to gain permanent benefit from a comparatively short course of sanatorium treatment. The case therefore should meet the following requirements:—

1. The constitutional evidences of disease should be slight.

2. The local involvement must not be extensive.

3. The previous history of the case and the family record should give grounds for believing that there are fair powers of natural repair.

4. The mental and moral condition of the patient should be such as will permit of a full acceptance of the educational and disciplinary advantages offered by a sanatorium, and the social position such as will allow of a continuance of a reasonably hygienic life after discharge.

The occurrence of a "pathological cure" is certainly not possible in all cases admitted, but an "economical cure" or arrest sufficient to allow the patient to return to the ranks of "the wage-earners" must be aimed at.

Briefly summarised, the most *favourable cases* will be found to be:—Adults, well developed, with no antecedent disease and of good family history, outdoor workers or engaged in an occupation which can be carried on under healthy conditions, free from alcoholic or other habits prejudicial to healthy life, country dwellers with good digestion and presenting but little evidence of constitutional derangement. A quiet, orderly, and hopeful individual makes the best patient. In females the continuance of menstruation is usually considered a favourable sign.

An acute onset in such a subject, even when accompanied by hæmoptysis or pleurisy, often gives way to a speedily established limitation and rapid restoration. Even when there is evidence of extensive involvement of lung, and considerable temperature, open-air treatment not infrequently works wonders. Many experienced observers consider that cases having an acute onset, when they react to open-air treatment, often do better than those in which the invasion was insidious and the disease has been allowed to progress along a chronic course. Cases with slight involvement of the larynx not infrequently do well. Generally speaking, however, it would be wise that such cases as these should be submitted to careful observation in the special hospital near town, and then if found suitable drafted on to the country sanatorium.

Generally speaking a case is considered favourable when there is absence of any marked rise of temperature, cachexia, anæmia, tendency to night sweats, dyspnœa, dyspepsia, or anorexia. A fairly slow and regular pulse of moderate tension is also a good sign. Hæmoptysis, unless it gives rise to marked anæmia, must not be considered a bar.

With an absence of constitutional symptoms the less marked the physical signs the better. Limited involvement of one apex with compensatory action of the other lung constitutes favourable local conditions. The more extensive the lesion and the more active the tubercular process, generally speaking, the less likely is the case to do well. Abundant sputum is often an unfavourable sign, but there may be absence of expectoration in some acute cases. The amount and character of the sputum affords evidence of but little value as to the patient's powers of restoration.

This is not the place to enumerate the various physical signs indicative of pulmonary disease nor the occasion for a discussion of their relative value. But in every case a thorough investigation of the whole chest should be made. The patient must be stripped to the waist and the chest carefully examined, both back and front. Unless this be done important conditions may sometimes be easily overlooked.

In every case of course the sputum will be examined, but comparatively little assistance can be gained from this alone in the formation of a reliable prognosis.

### Cases Unsuitable for Treatment in Public Sanatoria.

The following must be considered *unfavourable* cases for such sanatorium treatment as is at present available:—Patients below puberty and those in advanced life; town dwellers ill-developed and with a bad family history; those of unsuitable temperament; the alcoholic and reckless in habit; those engaged in dusty and confined occupations; and those who have suffered from former pulmonary or other diseases. A small or malformed chest is manifestly unfavourable. Frail, slender, excitable, irritable, neurotics generally prove bad subjects. Cases in which tubercle has become engrafted on a lung already the seat of chronic bronchitis and emphysema generally do badly. Even when there is no history of such preceding pulmonary involment cases in which bronchitic signs persist almost invariably give disappointing results. Young girls who break down at puberty often prove unfavourable subjects. All cases must be considered grave in which there is marked debility, a high degree of anæmia or characteristic cachexia. Persistent high temperature, sweats, uncontrollable cough, marked dyspnœa, intermittent attacks of pleurisy, anorexia, vomiting, dyspepsia, diarrhœa, insomnia, nervous irritability, despondency or melancholia must all be considered unfavourable manifestations. A rapid, irregular, unstable pulse, of low tension is very rightly considered a bad sign. Albuminuria and glycosuria are of evil omen.

Pregnant women are usually not welcome in a sanatorium; and after confinement the disease generally makes rapid progress.

Of course cases of phthisis in diabetic, alcoholic and syphilitic subjects must be passed over.

When tubercular infection follows post-influenzal debility the prognosis is grave. If the expectoration is profuse and contains fragments of lung tissue serious destructive changes are in progress. Fœtor of the sputum may sometimes be completely overcome by appropriate treatment.

The number of tubercle bacilli in the sputum affords no reliable guide to prognosis.

When there is marked laryngeal involvement, fistula in ano, or evidence of generalised tubercle, the outlook is serious.

As to the local signs, it is not well that too much dependence be placed upon them. In many cases even when but few indications of local mischief are apparent, the general condition will immediately mark the case as an unfavourable one; and on the other hand, although there may be extensive cavitation and widespread disease of one or even of both lungs, yet when the general condition is fairly good, such cases are often greatly benefited by outdoor treatment. It is well to realise that the old pathological and clinical groupings dependent on so-called "stages" are not to be relied upon absolutely in forming a prognosis or as indicating degrees of

"curability." In selecting cases for treatment I am more and more inclined to consider physical signs of subordinate importance. Although, roughly speaking, we may say the more extensive the disease the more serious the case, this often requires serious qualification. A reliable conclusion can only be arrived at by considering the *whole* case.

But at best our data for the formation of an exact prognosis is far from complete. There is need for the careful collection and judicious collation of the results of modern treatment. Many of our old ideas will have to be recast and reset. We need extension of our knowledge regarding the variations in the virulence of the tubercle bacillus and its toxins when developing within the human body. More light must be thrown on the conditions favouring or retarding the growth of the parasite on human soil. The influence of mixed infection is not sufficiently allowed for in clinical work. And much remains to be done in collecting evidence as to the permanency of the beneficial effects of hygienic treatment.

### Auxiliary Forces in the Combat with Consumption.

I have already insisted that the provision of sanatoria as we now know them forms but a part of a much more comprehensive campaign. It is only possible here just to hint at the auxiliary forces which must be brought into action before we may hope to claim conquest over consumption.

The benefits of sanatorium treatment have clearly demonstrated the evils of *insanitary dwellings.* Conditions exist in many homes, and they are not confined to the poor only, which render hygienic life impossible; tubercular infection is facilitated, the virulence of the tubercle bacillus is maintained, and it may be even increased, and a lowering of the powers of resistance brings about marked vulnerability of tissues.

The imagination of the people associates a sanatorium with definite disease. The day will come when we shall realise the necessity of providing properly situated and suitably equipped establishments for those prone to depart from the normal standard of health. *Sanatoria* are urgently needed as *prophylactic institutions.*

As a supplement to the sanatoria for early cases of actual consumption which are now being established in various parts of the country *convalescent homes for* cases of *arrested consumption* are sadly required. At present the stay in a public sanatorium is necessarily restricted. As I have already shown, the exigencies of the case often necessitate discharge when but the beginnings of arrest have been secured. To establish a satisfactory restoration and firmly consolidate those tissue changes which constitute the physical basis of what we are pleased to call a "cure," time is a necessary factor. Much might be accomplished if the convalescent consumptive could, before returning to take a place in the ranks of busy workers, obtain an extended continuance of open-air life under the best conditions. At present most convalescent homes very rightly close their doors to consumptives. An extension of cottage homes might prove of much benefit. After a practical experience of the advantages of strict hygienic treatment the residence of a judiciously selected case in a farmhouse or village home might do much to bring about a more rational state of life than unfortunately exists in many country districts.

It is necessary that more efficient measures should be taken for the *after-care* of cases who have undergone sanatorium treatment.

It may also be pointed out that when the bread-winner ceases to be a wage-winner and becomes an inmate of a sanatorium, he not infrequently has to leave his wife and children practically destitute. Here is an opportunity for the benevolent.

A plea must also be made for the establishment of *seaside sanatoria for children.* The various seaside homes, usually available only during the summer months, accomplish a noble work in furnishing conditions necessary for healthy development. Undoubtedly they serve to strengthen the powers of resistance of many only too prone to become a prey to tuberculosis. But there is much need for a further growth of sanatoria designed and conducted with the definite aim of serving as prophylactic stations for those children who from inherited or acquired tendencies are predisposed to tuberculosis. While for many phthisical adults the sea coast presents features which are unfavourable; for many, and indeed for most children it certainly proves beneficial.

But for many cases, unfortunately, sanatorium treatment is at present out of the question. For these it is desirable that hygienic measures should at least be attempted in their *home treatment.* Even in a large city like London much benefit has accrued from adopting an open-air regime. The advantages of *notification* are particularly manifest in respect of these cases. A judicious Medical Officer of Health is enabled, by providing measures for *disinfection* and in other ways not only to render the patient much service, but to take steps to arrest the spread of the affection.

*Homes for advanced cases* are urgently needed where the latter days of incurables may be rendered as bright and happy as may be possible. In many instances it is only by the isolation of such cases that relatives and neighbours can be saved from the serious risk of infection.

The increasing prevalence of phthisis among the insane urgently calls for the establishment of properly-constructed hospitals and sanatoria for the isolation and rational treatment of these particularly distressing cases.

It is necessary also to point out that, unfortunately for many, sanatoria treatment can never be expected to refit the sufferer so as to renew the struggle for existence on the same plane as the healthy co-worker. Sooner or later we may be wise enough to provide suitable "garden towns" or "*colonies*" for the maimed and handicapped consumptive.

For long humanity has laboured, and men and women and little children have struggled and died with the blighting shadow of this fell scourge ever upon them; but now, at last, the scales of ignorance seem to have fallen from our eyes, and though we still see as through a glass darkly, yet a measure of relief has come, and ties of tradition and fetters of authority are broken; superstition and ignorance are being cast aside and men and women are answering to the call of hope :—

"Come forth into the light of things,
Let Nature be your teacher."

